# Combined-therapeutic strategies synergistically potentiate glioblastoma multiforme treatment *via* nanotechnology

**DOI:** 10.7150/thno.40298

**Published:** 2020-02-10

**Authors:** Jun Yang, Zhuyan Shi, Ruiyuan Liu, Yanyue Wu, Xin Zhang

**Affiliations:** 1 State Key Laboratory of Biochemical Engineering, Institute of Process Engineering, Chinese Academy of Sciences, Beijing 100190, PR China; 2 University of Chinese Academy of Sciences, Beijing 100049, PR China

**Keywords:** glioblastoma, combined therapy, nanotechnology, drug delivery

## Abstract

Glioblastoma multiforme (GBM) is a highly aggressive and devastating brain tumor characterized by poor prognosis and high rates of recurrence. Numerous therapeutic strategies and delivery systems are developed to prolong the survival time. They exhibit enhanced therapeutic effects in animal models, whereas few of them is applied in clinical trials. Taking into account the drug-resistance and high recurrence of GBM, combined-therapeutic strategies are exploited to maximize therapeutic efficacy. The combined therapies demonstrate superior results than those of single therapies against GBM. The co-therapeutic agents, the timing of therapeutic strategies and the delivery systems greatly affect the overall outcomes. Herein, the current advances in combined therapies for glioblastoma *via* systemic administration are exhibited in this review. And we will discuss the pros and cons of these combined-therapeutic strategies *via* nanotechnology, and provide the guidance for developing rational delivery systems to optimize treatments against GBM and other malignancies in central nervous system.

## Introduction

Glioblastoma multiforme (GBM) is a primary malignant brain tumor of the central nervous system (CNS) with poor prognosis and high mortality 1, 2. The median survival rate is around 12-18 months, and the long-term survival time is less than 5 years 3, 4. The current clinical treatments for GBM consist of tumor resection, radiotherapy and chemotherapy. Maximal tumor resection improves the overall survival in GBM. But it is difficult to distinguish tumor from normal brain tissues. Several intraoperative technologies, such as 5-aminolevulinic acid (ALA) and fluorescein (FLCN) guided resection, facilitate the identification of tumor and simultaneous preservation of neurologic function 5, 6. They significantly improve the gross total resection with standard surgery from 36% to over 64% in high-grade gliomas (HGG). However, the infiltrative nature of GBM raises the challenge to realize a complete resection. Furthermore, the extent of resection must be balanced with the brain function 7. The introduction of Stupp regimen and modified Stupp extends the median survival of the patients, whereas the overall survival is still poor 8-10.

Besides the standard therapy, the therapeutic strategies like gene therapy, immunotherapy, phototherapy and thermotherapy, have been applied for anti-glioma treatments 11-15. They exhibit enhanced therapeutic effects in animal models, but few of them is applied in clinical trials. Since the GBM is a complex disease with intricate mechanisms in their growth, progression and invasion processes, accumulating evidences indicate that single therapeutic strategy tends to result in drug resistances and tumor cell tolerance, which finally leads to tumor recurrence and metastasis 16, 17. Therefore, combined-therapeutic strategies with various mechanisms of agents should be able to overcome these problems.

**Figure 1:**
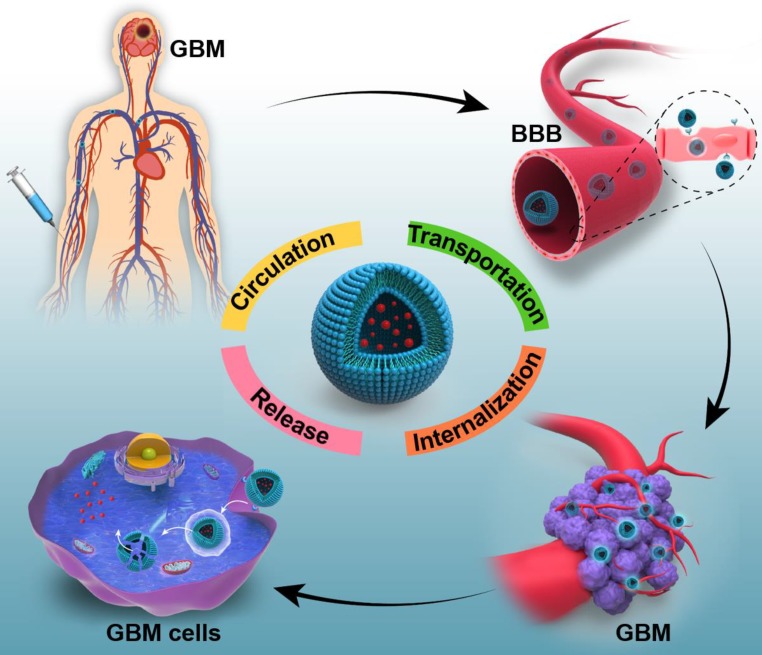
The schematic illustration exhibits the challenges of drug delivery *via* systemic administration for glioblastoma therapy. They generally include: (1) long circulation in the blood; (2) effective transportation across the BBB; (3) efficient internalization into GBM cells and (4) controlled drug release in the tumor cells.

However, the current therapeutic agents have some critical problems, such as short half-life in circulation, hard transportation to the diseased areas and difficulty of controlled releasing the diverse drugs in the corresponding sites. These problems result in insufficient accumulation of therapeutic drugs in the tumor cells, and prevent adequate destruction of malignant tumors 18, 19. Therefore, efficient drug delivery will be very critical and important for effective therapy 20. For a typical cancer treatment, a five-step CAPIR cascade in drug delivery process is presented by Shen's group 21. It includes circulation in the blood, accumulation in the tumor, penetration into tumor tissue, internalization into tumor cells and intracellular drug release. High efficiency at every step is very important to ensure the high therapeutic efficiency of the whole treatment. Furthermore, in view of the location of glioblastoma, the specific blood-brain barrier (BBB) also constructs a major and critical obstacle for the drug delivery 22, 23. It is estimated that over 98% of the small molecular drugs and almost 100% of large molecular drugs cannot cross the BBB 24, 25. Given this, the challenges of drug delivery in the GBM treatment should be composed of the following steps: long circulation in the blood, effective transportation across the BBB, efficient internalization into glioma cells and controlled drug release in the cells (Figure 1). All of these steps are the key factors to ensure sufficient therapeutic agents accumulating in GBM cells.

To overcome these physiological barriers in GBM treatments, nanotechnology has been explored to solve these problems and potentiate the therapeutic effects. These delivery systems can be classified as liposomes, polymer nanoparticles, lipopolymer nanoparticles, dendrimer nanoparticles, hybrid nanoparticles and so on 26-29. Firstly, the diversity of these biomaterials can enable loading of various therapeutic agents simultaneously. Secondly, these nanodelivery systems can be modified with specific targeting, which shows the guidance to BBB and the GBM cells. The reported brain tumor targeting moieties include transferrin receptor (TfR), angiopep-2 peptide, TAT peptide, RGD peptide, chlorotoxin and so on 30-32. The modified nanodelivery systems can transport across the BBB by receptor-mediated endocytosis, adsorptive-mediated endocytosis and carrier-mediated transport 33. These targeting modifications greatly improve the brain tumor targeting and reduce the side effects to normal tissues. Thirdly, stimuli-sensitive responses are introduced into delivery systems to ensure the maximal drug retention at the desired sites, such as pH, ROS, enzyme, light and thermal responses 34. These strategies with nanotechnology prompt the drug accumulation in brain tumors. Intensive advances have exhibited the effective and promising outcomes for glioblastoma therapy these decades, but few of them is applied in clinical trials until now. Herein, in this review, we will focus on the combined-therapeutic strategies with diverse delivery systems, investigate the current combined strategies with improved therapeutic effects, and discuss the future guidance for glioblastoma treatments and other CNS diseases' therapies.

## Combined therapies against glioblastoma

The complexity of glioblastoma multiforme motivates researchers to develop various therapeutic strategies. Comparing with single therapy, combined therapies have emerged new issues to concern. The choice of the therapeutic strategy for combination is the first key factor. It contains the selection of combined methods and the therapeutic agents, and synergism of these strategies. The delivery systems based on the therapeutic agents are the second important issue to concern. These systems of combined therapies are required to load the multiple therapeutic agents and deliver them to the corresponding targeted sites for synergistic treatments. Herein, various combined-therapeutic strategies for anti-glioma treatments are presented in the following sections. And the merits of the current delivery systems are discussed, which should inspire and guide to develop optimized delivery platforms for further clinical applications.

### Combined chemotherapies

Chemotherapy was the most common treatment for glioblastoma. Single chemotherapy facilitated the drug resistance of the tumor after a certain period, which greatly hindered the successful treatment of GBM. Combination with diverse mechanisms of anticancer drugs should improve the GBM therapy, and some efforts had entered into clinical trials 35-38.

However, due to the physiological barriers of glioblastoma, some clinical trials on the combination therapies did not show survival advantages and failed to obtain the desirable outcomes 39-41. Hence, numerous delivery systems were developed to overcome these challenges and obtained better therapeutic effects 42, 43. For example, Guo et al. utilized a liposome (LP), which was composed of egg yolk phosphatidylcholine (EPC), cholesterol (Chol) and 1,2-distearoyl-*sn*-glycero-3-phosphoethanolamine-N-[methoxy(polyethylene glycol) 2000] (DSPE-PEG2000), to separately encapsulate the tumor necrosis factor-related apoptosis-inducing ligand (TRAIL) and doxorubicin (DOX) 44. The median survival time of the combination group (DOX-LP+TRAIL-LP) extended to 48 days, compared with the single groups TRAIL-LP (32 days) and DOX-LP (39 days). The combined chemotherapies showed improved therapeutic effects in the intracranial U87MG-bearing mice. The proportion of the drugs was easy to adjust by the detached drug load, but the synergism effects of these anticancer drugs *in vivo* might be hard to control.

To increase the drug accumulation in the brain tumors, active targeting was introduced into the nanosystems to enhance the drug concentration in tumor sites and reduce the systemic toxicity 45-47. Erica Locatelli et al. developed a targeted delivery nanoparticle (Ag/Ali@PNP-Cltx-^99m^Tc) based on the poly(lactic-*co*-glycolic acid)-block-polyethylene glycol (PLGA-*b*-PEG) copolymer 48. The nanosystem conjugated with chlorotoxin (Cltx), which expressed the specific binding to matrix metalloproteinase-2 (MMP-2), a receptor overexpressed on the brain cancer cells. The *in vivo* biodistribution data exhibited that the targeted nanosystem Ag/Ali@PNP-Cltx-^99m^Tc significantly increased drug concentration in tumors. The enhanced drug accumulation obviously reduced the tumor size *in vivo*. The current approved anticancer drug temozolomide (TMZ) had been reported to associate with the introduction of O^6^-methylguanine-DNA methyltransferase (MGMT), which increased TMZ-resistance in chemotherapy 49-54. Sang-Soo Kim et al. developed a cationic liposome (scL-p53) encapsulating the tumor suppressor gene p53 plasmid DNA to sensitize the cancer cells to TMZ 55. This nanocomplex was modified with an antitransferrin receptor (TfR) single chain antibody fragment (scFv) to target the tumor cells. The combination of scL-p53 with TMZ obviously down-modulated the MGMT expression by 95% for 24 h *in vivo*. The mice treated with cycle administration of TMZ finished at day 45, whereas 63.6% of the mice were still alive when treated with this nanosystem. These studies demonstrated that the active brain targeting significantly enhanced the drug concentration in tumors with the same doses in comparison with the non-targeted systems.

**Figure 2:**
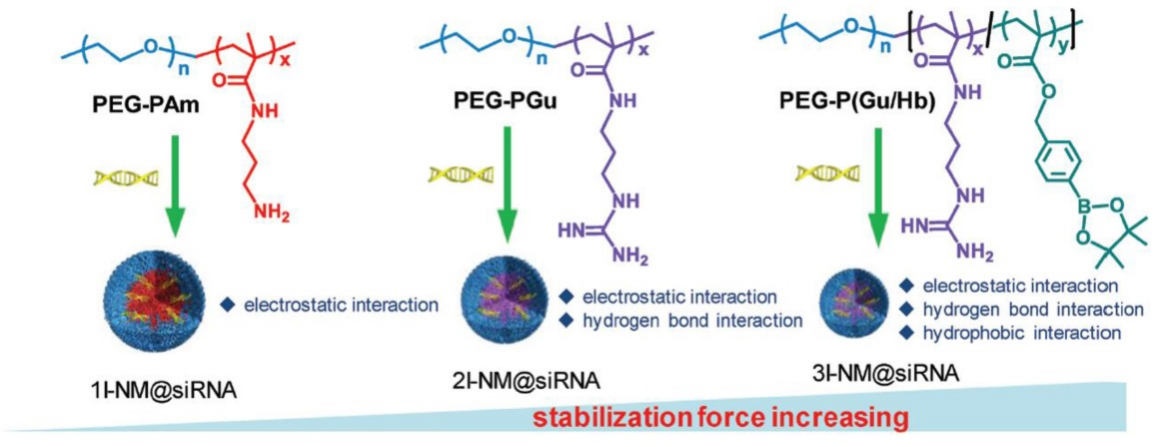
The schematic illustration of the formation of 1I-NM@siRNA, 2I-NM@siRNA and 3I-NM@siRNA utilizing different polymers to generate different numbers of stabilizing interactions. Adapted with permission from 62. Copyright 2019 WILEY‐VCH Verlag GmbH & Co.

Active brain targeting ensured the guidance of drug accumulation in tumor tissues, and the effective release of therapeutic drugs in tumor cells was also critical for efficient treatments against GBM 56. Hence, plenty of stimuli-sensitive drug delivery systems were designed based on the microenvironment of GBM, including pH, ROS, enzyme, and so on 57-59. For example, the anticancer drug doxorubicin (DOX) could potentiate the antitumor effect of tumor necrosis factor-related apoptosis-inducing ligand (TRAIL), which expressed by pORF-hTRAIL. Inspired by these, Jiang's group designed a targeting dendrigraft poly-L-lysine (DGL)-based nanosystem (DGDPT/pORF-hTRAIL) with pH-response to carry the chemotherapeutic drug DOX and the gene agent pORF-hTRAIL for anti-glioma therapy 60, 61. This nanosystem was modified with peptide HAIYPRH (T7), a transferrin receptor-specific peptide, for brain tumor cells targeting. The target significantly increased the drug accumulation in tumors *in vivo*. The median survival of DGDPT/pORF-hTRAIL was extended to 57 days, in comparison with 34 days for the free DOX. More importantly, the dose of the anticancer drug DOX in DGDPT/pORF-hTRAIL was less than 0.35 mg/kg mice, whereas the dose of free DOX was 5 mg/kg mice. This nanosystem greatly increased the pharmaceutical effect of the DOX and reduced the side toxicity to normal tissues. Also, Shi's group designed a nanoparticle (3I-NM@siRNA) for combined siRNA delivery, which was stabilized by electrostatics, hydrogen bonds and hydrophobic interactions (Figure 2) 62. The angiopep-2 targeting decorated 3I-NM@siRNA was prepared with poly(ethyleneglycol)-block-poly[(N-(3-methacrylamidopropyl)guanidinium-co-4-(4,4,5,5-tetramethyl-1,3,2-dioxaborolan-2-yl) benzyl acrylate)] (PEG-b-P(Gu/Hb))/ Ang-poly(ethylene glycol)-block-poly(N-(3-methacryl amidopropyl) guanidinium) (Ang-PEG-*b*-PGu). The nanoparticles could trigger drug release under ROS environment that enriched in the cancer cells. Treatment with Ang-3I-NM@(siPLK1+siVEGFR2) significantly increased the median survival time to 36 days, which was longer than the mice treated with 3I-NM@(siPLK1+siVEGFR2) (18 days), Ang-3I-NM@(siPLK1) (24 days) and Ang-3I-NM@ (siVEGFR2) (26 days).

**Figure 3:**
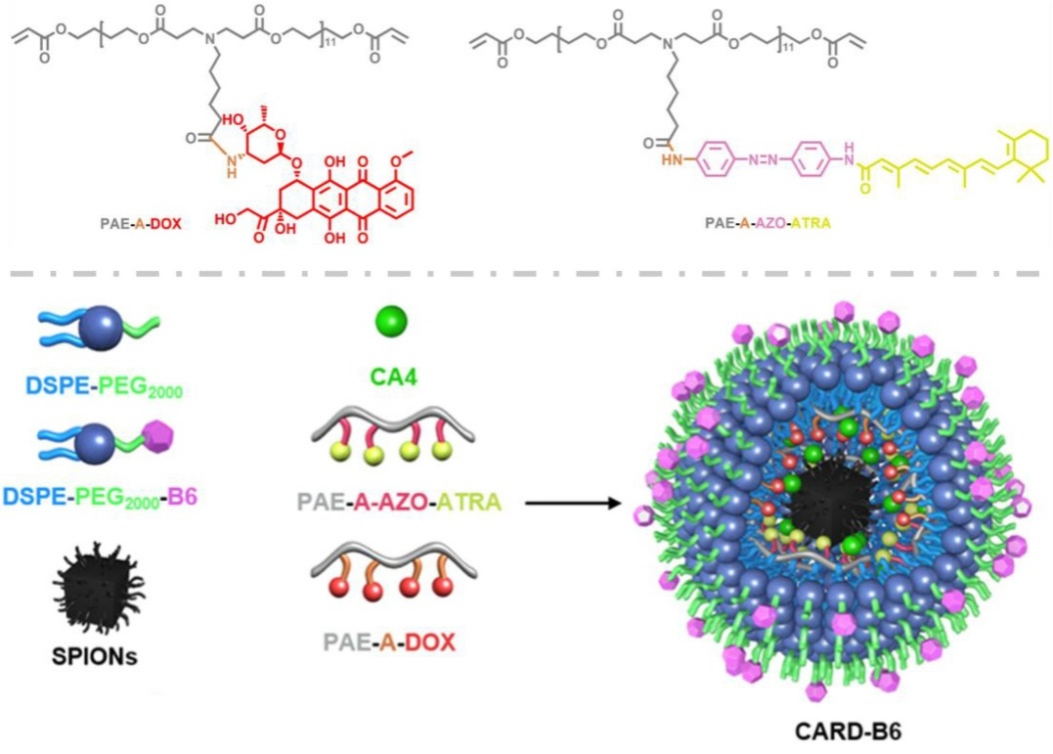
The structural composition and preparation route of the multifunctional nanoparticles CARD-B6. Adapted with permission from 67. Copyright 2017 WILEY‐VCH Verlag GmbH & Co.

Numerous researches detected that the available drug therapies were dissatisfied because they failed to penetrate into depth of the tumor tissues and kill the brain cancer stem cells (BCSCs), which was the most responsible factor for tumor recurrence 63-65. Intensive studies had been arisen to inhibit the growth of cancer stem cells for effective treatments to glioblastoma 66. Lu et al. in our group designed a brain targeting and multifunctional nanoparticle (CARD-B6) to synergistically aim at both the glioblastoma cells and glioblastoma stem cells (GSCs) (Figure 3) 67. In this nanosystem, acid-sensitive poly(β-amino ester) (PAE) was utilized to load the antiangiogenic drug combretastain A4 (CA4), and triggered to release the cargo in the GBM microenvironment. The azobenzene (AZO) bond in the PAE, rapidly broken up in the hypoxic GSCs, was applied to conjugate with all-trans retinoic acid (ATRA), which could induce the GSCs differentiation into glioblastoma cells. The amide bond in the PAE was conjugated with anticancer drug doxorubicin (DOX) for controlled release in tumor cells. The designed CARD-B6 could spatiotemporally trigger to deliver these drugs to their corresponding target sites. And the survival time of the CARD-B6 treated mice significantly enhanced, with 60% survivals *vs.* 0% for all the other control groups.

**Figure 4:**
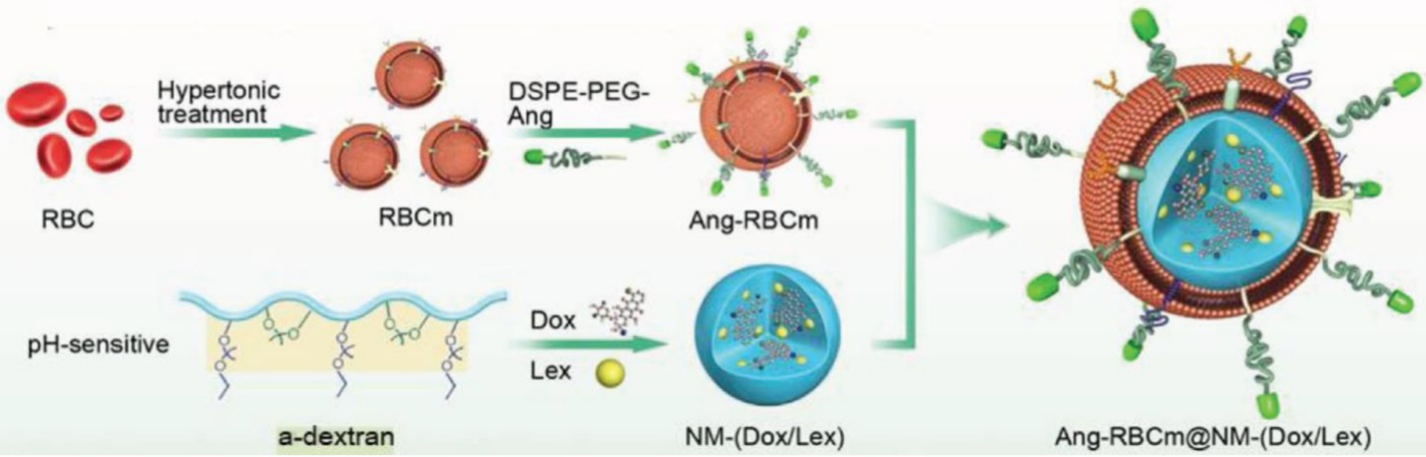
The schematic illustration of the formation of the biomimetic nanomedicine (Ang-RBCm@NM-(Dox/Lex)). Adapted with permission from 74. Copyright 2018 WILEY‐VCH Verlag GmbH & Co.

Recently, biomimetic nanosystems were exploited for drug delivery applications, including the utilization of red blood cell membranes, tumor cell membranes, platelet membranes and etc. 68-73. They had good biocompatibility and avoided immunogenicity. For instance, Shi's group designed a biomimetic nanomedicine (Ang-RBCm@NM-(Dox/Lex)) by functionalizing with red blood cell membranes (RBCms) (Figure 4) 74. This nanosystem was equipped with angiopep-2 at the surface of red blood cell membranes. The target had high affinity for the low density lipoprotein receptor-related protein (LRP) receptor, which was overexpressed on both the endothelial cells of the BBB and glioblastoma cells. A pH-sensitive a-dextran that coloaded the anticancer drug doxorubicin (Dox) and lexiscan (Lex), and encapsulated in the RBCm, could transiently open the BBB to enhance the permeability of the nanomedicine Ang-RBCm@NM-(Dox/Lex). The Ang-RBCm@NM-(Dox/Lex) exhibited a longer blood circulation time, with the half lifetime of 9.3 h, whereas the circulation time of NM-(Dox/Lex) without RBCm camouflage was only 2.4 h. And the biodistribution of Ang-RBCm@NM-(Dox/Lex) in the orthotopic brain tumor was about 3.5-fold higher than that of NM-(Dox/Lex). The biomimetic nanomedicine enhanced the median survival time to 38 days, in comparison with RBCm@NM-(Dox/Lex) (28 d), Ang-RBCm@NM-Dox (25 d), NM-(Dox/Lex) (22 d), free Dox (22 d) or PBS (18 d) treated groups.

Chemotherapy was the most widely studied treatment for anti-glioma therapy. Mounting researches were reported from the non-targeting and non-sensitive delivery systems to the active targeting and stimuli-sensitive systems. Intelligent multifunctional delivery systems were designed in dependence on the specific characteristics of the GBM. They exhibited better drug accumulation and longer survival time for GBM treatments. However, GBM was a complex disease. The initiation, growth and procession of the GBM were not completely understood until now. Hence the therapeutic drugs might not involve in every critical point of the whole treatments, and the designed drug delivery systems based on existing knowledge of GBM could not completely eliminate the GBM. Moreover, most of the biomaterials utilized in these systems were not approved by FDA, which aggravated difficulties for the clinical translations.

Biomimetic drug delivery systems offered new opportunities to mimic the biological vehicles. The naturally derived cell membranes decorated the drug delivery systems, and brought longer circulation and better biocompatibility for these nanosystems. However, they also generated new problems, like the preparation operations and the cost of the biological products, the strictness production conditions for the clinical translations and applications.

### Combined chemotherapy with radiotherapy

Radiotherapy was one of the clinical treatments for GBM, and amount of efforts had been made to increase the efficacy 4. However, the invasive tumor growth of the glioblastoma limited the radiotherapy's efficacy and induced tumor recurrence. Since the combination of radiotherapy with chemotherapy turned into the standard treatment strategy to inhibit GBM recurrence 75-78, the radiotherapy concomitant with free temozolomide or carmustine entered into clinical trials. But the regimens were still insufficient for the requirements 79.

**Figure 5:**
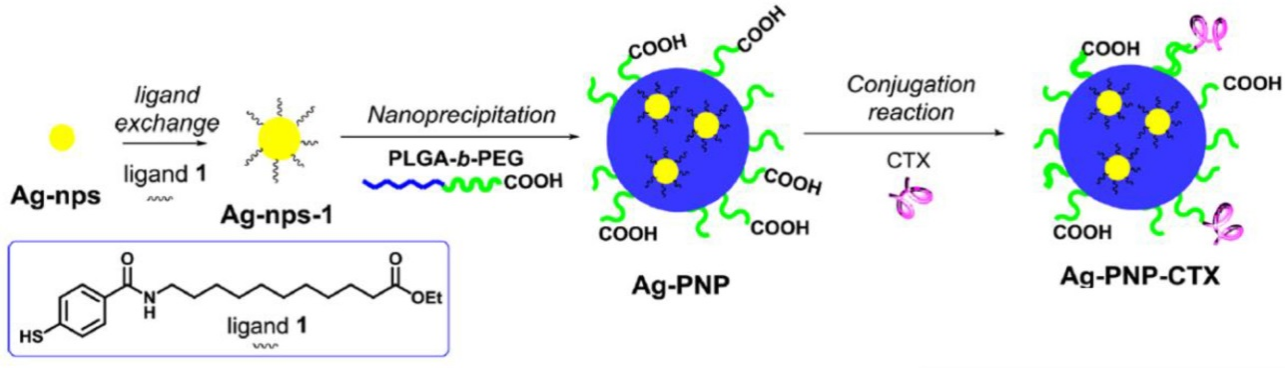
Schematic procedure for the synthesis of chlorotoxin-nanovectors (Ag-PNP-CTX). Adapted with permission from 81. Copyright 2016 American Chemical Society.

Hence, delivery systems with nanotechnology were exploited for the combined therapies to maximize the therapeutic effects. Shi et al. developed an integrin targeting ^177^Lu radiolabeled 3PRGD_2_ (a dimeric RGD peptide with 3 PEG_4_ linkers) for radiotherapy 80. The cellular uptake of ^177^Lu-3PRGD_2_ in U87MG tumors was about 6%ID/g for 1 h. The combination with the anti-angiogenic agent Endostar exhibited significant tumor inhibition in comparison with the control group. Later, Matteo Tamborini et al. reported that combining the chlorotoxin-nanovectors and radiation could exhibit a synergistic effect for *in vivo* GBM growth (Figure 5) 81. Chlorotoxin, a selective target and anticancer drug, was conjugated on poly(lactic-co-glycolic acid) (PLGA) nanoparticles (PNP). Low dose of radiation (2 Gy) rendered the brain extracellular matrix permeable and facilitated the accumulation of the nanovectors (Ag-PNP-CTX). The combination therapy significantly inhibited approximately 50% tumor growth in U87MG-bearing mice.

As the clinical standard treatment, radiotherapy often resulted in the modest improvement for glioblastoma treatments without reducing the quality of life or cognition. From the available better treatments, radiotherapy with concomitant chemotherapy was the current standard of care. However, the reported clinical trials regarding the combination of radiotherapy and chemotherapy exhibited diverse results. For instance, the addition of bevacizumab to radiotherapy-temozolomide did not improve the survival against GBM in phase 3 study 79. And sorafenib combined with radiotherapy-temozolomide exhibited severe side effects in phase 1 study 82. These might be due to the mechanisms of these drugs or the insufficient drug concentrations in the tumors, which counteracted the therapeutic effects. Hence, the definition of tumor margins, the choice of the chemotherapeutic drugs and drug delivery systems were very important for the regimen.

### Combined chemotherapy with phototherapy

Phototherapy was composed of the photothermal therapy (PTT) and the photodynamic therapy (PDT), which was a promising non-invasive strategy for cancer treatments. The former one utilized the photothermal agents to generate heat and kill tumor cells under light absorption. And the latter one produced cytotoxic reactive oxygen species, such as singlet oxygen (^1^O_2_), free radicals or peroxides, to induce cell death 83-85. Nanotechnology assisted the phototherapy into further applications since most of the photosensitizers and photothermal agents were hydrophobic and had low tumor selectivity 15, 86-89.

**Figure 6:**
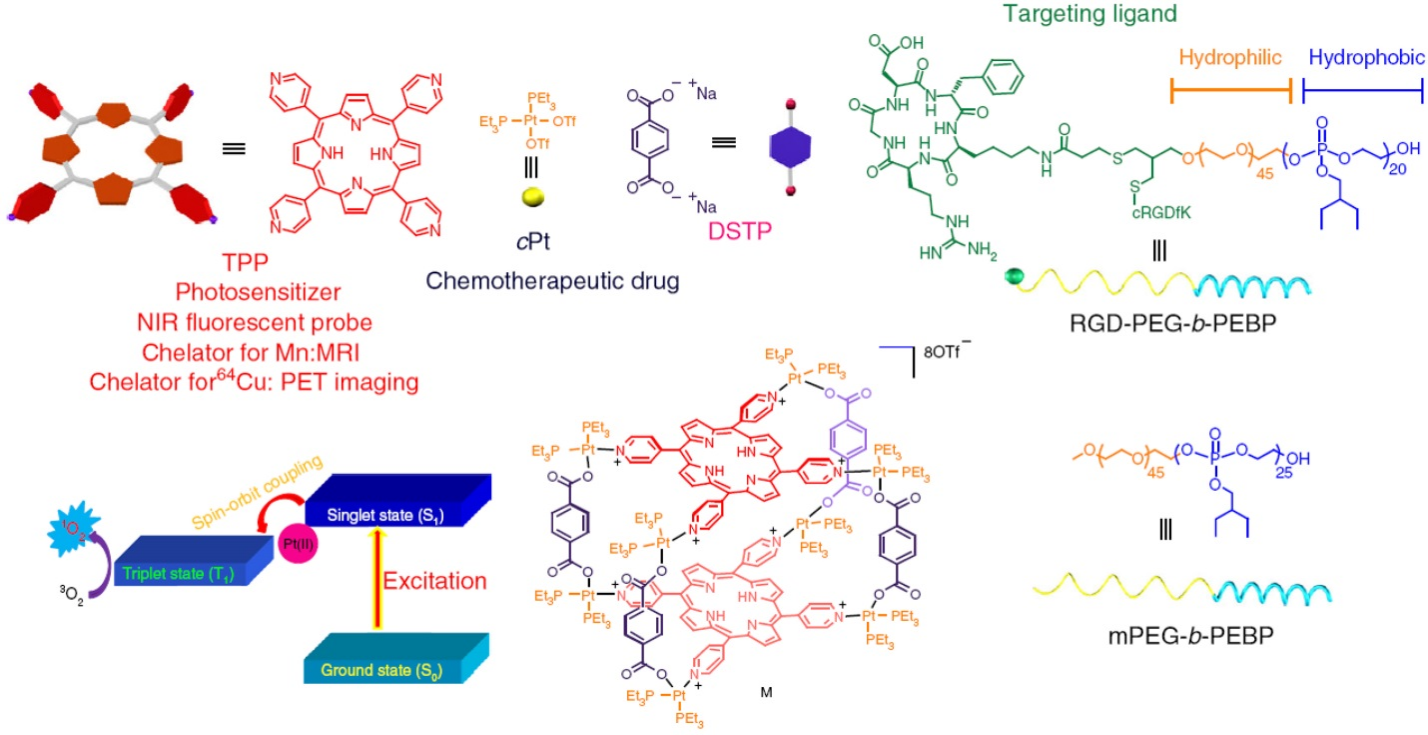
Schematic diagrams of the MNPs served as a multifunctional theranostic platform. The structures of TPP, cPt, DSTP, M, mPEG-*b*-PEBP and RGD-PEG-*b*-PEBP were illustrated. Adapted with permission from 94. Copyright 2018 Springer Nature.

However, phototherapy alone could not kill the tumor cells entirely due to the uneven light distribution and hypoxic condition in the tumor tissues, and it was easy to induce local recurrence and distant metastasis, especially for glioblastoma 90, 91. Hence, combination of the phototherapy with chemotherapy could highlight the potential approaches for malignant glioblastoma therapies 92, 93. For instance, Yu et al. designed an organoplatium (II) metallacage (M) coordinated by photosensitizer 5, 10, 15, 20-tetra(4-pyridyl)porphyrin (TPP), therapeutic drug cis-(PEt_3_)_2_Pt(OTf)_2_ (cPt), and disodium terephthalate (DSTP) 94. The metallacage self-assembled with mPEG-*b*-PEBP and RGD-PEG-*b*-PEBP to form metallacage-loaded nanoparticles (MNPs) for long blood circulation and active targeting (Figure 6). The MNPs exhibited superior anticancer results against U87MG with no recurrence after single injection, which attributed to the synergistic photochemotherapy.

The inherent proteins were applied as drug carriers due to their superior biocompatibility. Liu's group utilized the human serum albumin (HSA) as the carrier to conjugate with the photosensitizer chlorin e6 (Ce6) and self-assemble with the anticancer drug paclitaxel (PTX) 95. An acyclic Arg-Gly-Asp (cRGDyK) peptide, targeting to ανβ3-integrin overexpressed on tumor angiogenic endothelia, was chosen as the active targeting (HSA-Ce6-PTX-RGD). The accumulation of the nanoparticles was about 2.4 times higher than the non-targeting one *in vivo*. The survival time after the combination therapy prolonged to 40 days without a single death, while the mice treated with control groups exhibited lifetimes about 15-30 days.

Gold nanoparticles were the attractive photothermal agents for cancer therapies due to their strong absorbance of NIR light and higher heat conversion efficiency 96-99. Yongwei Hao et al. developed a tumor-targeting hybrid nanosystem by modifying Au on the surface of docetaxel (DTX)-loaded poly(lactide-co-glycolide) (PLGA) nanoparticles (ANG/GS/PLGA/DTX NPs) 100. The U87MG cells treated with nanosystem showed a striking temperature increase for 12.3°C when exposed to 808 nm irradiation. And the tumor inhibition rate of the ANG/GS/PLGA/DTX NPs under irradiation was about 70%, which was the highest among all of the control groups for *in vivo* studies.

**Figure 7:**
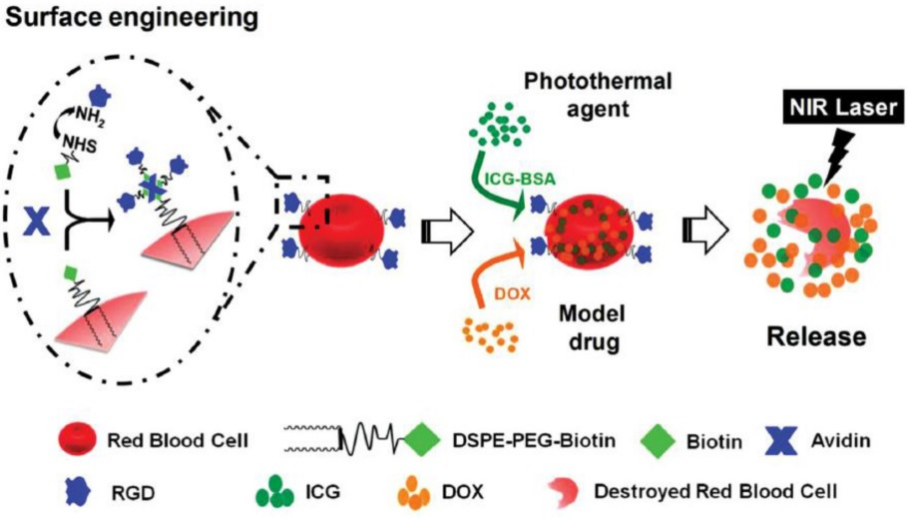
Schematic illustration for the preparation of the RBC-based system (IB&D@RBC-RGD) and the remotely controlled drug release under the NIR laser. Adapted with permission from 103. Copyright 2015 WILEY‐VCH Verlag GmbH & Co.

The biomimetic drug delivery systems coated with native cell membranes also attracted researchers' attentions to fight against cancers with phototherapy 101, 102. Liu's group exploited a RBC-based system (IB&D@RBC-RGD) with a tumor angiogenesis targeting (Figure 7) 103. Anticancer drug DOX and photothermal agent indocyanine green-bovine serum albumin nanocomplexes were co-loaded into the modified RBCs. With deep penetrability of near-infrared (NIR) light, the IB&D@RBC-RGD significantly released the cargos and enhanced cytotoxicity against U87MG and 4T1 cells.

Phototherapy for GBM was restricted due to the limited tissue penetration and photosensitizers' delivery. Nanosystems were applied in photosensitizers' delivery for targeting transportation and reducing the non-specific accumulation in normal tissues. However, due to the highly invasive growth of GBM, there usually had tumor recurrence and metastasis after phototherapy. Combined therapies by nanotechnology maximized the anti-glioma efficacy, which were attributed to the significant increase of chemotherapeutic drugs and photosensitizers/photothermal agents in the tumor cells. And these were confirmed by a growing amount of studies. But the challenges for this combined-therapeutic strategy against GBM still remained, including the ratio of the therapeutic drugs and photosensitizers/photothermal agents, the drug-to-light interval and the synergistic interaction of the therapeutic methods. All of these should greatly affect the anti-glioma effects and need comprehensive studies in future.

### Combined chemotherapy with immunotherapy

Immunotherapy had received tremendous attentions in the treatment of cancers in the decades 104. It could activate the body's own immune systems and induce the specific immune responses with tumor antigens to eliminate the tumor cells 105, 106. Most importantly, it had the potential for long-term reduction of cancer metastasis and recurrence 107. The immunotherapeutic strategies included the cancer vaccines, monoclonal antibodies, oncolytic virus, engineered T-cells and immunomodulation 11, 107, 108. However, the major challenges of immunotherapy for glioblastoma were the lack of specific tumor antigens, limited immunogenicity of the cancer cells and the immunosuppressive environment of the tumors 109-111. Intensive studies had been reported to promote the specific immune responses and exhibit effective immunotherapy for glioblastoma 112, 113.

The epidermal growth factor receptor variant III (EGFRvIII), an oncogenic variant of the EGFR, expressed in 20-30% of all glioblastoma 114. It was exploited to cancer vaccine (Rindopepimut) and showed an encouraging progression-free survival in clinical trials 115, 116. However, the combination of Rindopepimut and temozolomide in phase 3 trial did not show significant difference in overall survival for EGFRvIII-expressing glioblastoma 117. This might be due to inappropriate combination of the drugs, which counteracted the immunological and chemical effects. And the unspecific tumor antigens, which were not sufficient to cover the heterogeneous brain tumors, also influenced the therapeutic effects of GBM.

**Figure 8:**
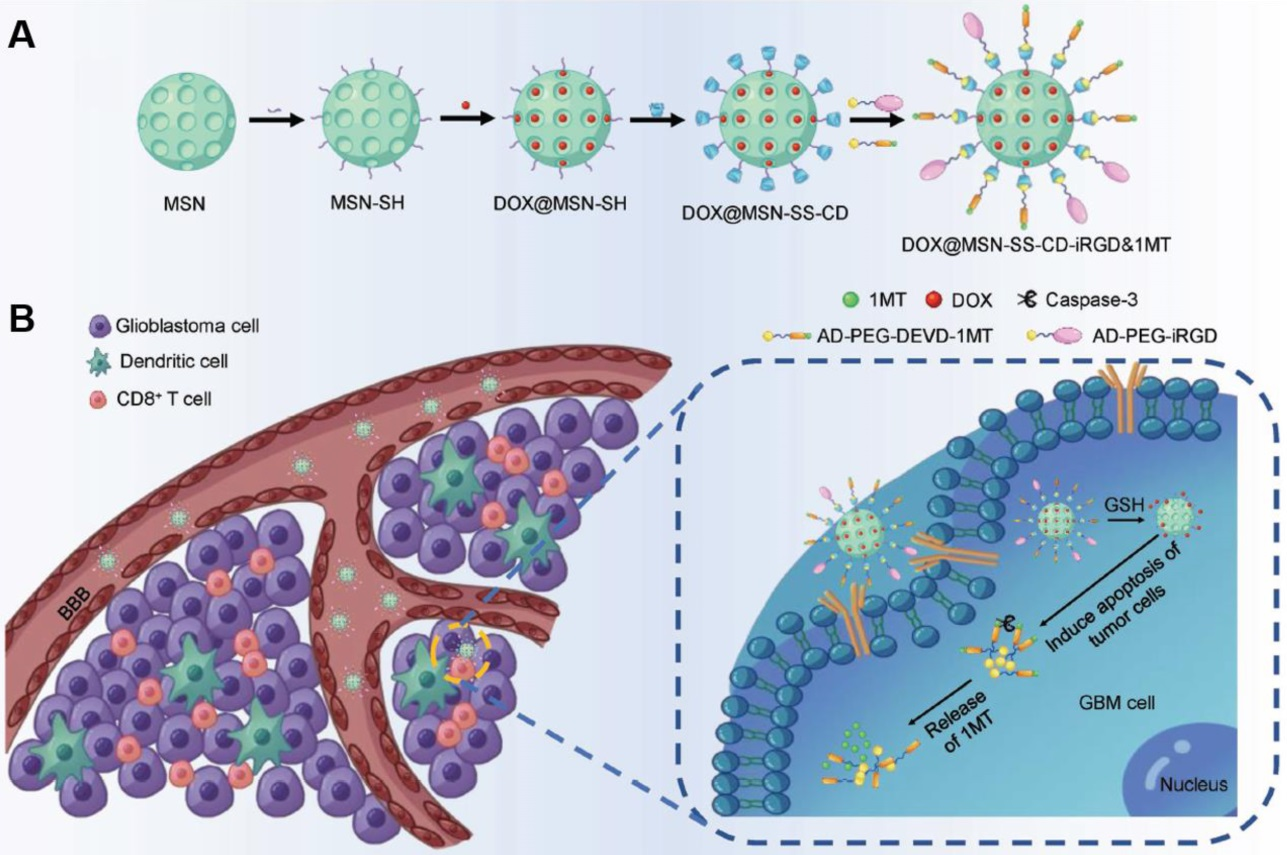
A) The synthetic route of the iRGD-modified silica nanoparticles DOX@MSN-SS-iRGD&1MT. B) The illustration of DOX@MSN-SS-iRGD&1MT showed active targeting and drug release. Adapted with permission from 119. Copyright 2018 WILEY‐VCH Verlag GmbH & Co.

The immune checkpoint was a costimulatory factor for regulating the antigen recognition in the process of immune responses. The inhibition of the checkpoint could enhance the anticancer immune activation 118-120. Combination of immune checkpoint with chemotherapy could stimulate the tumor immunity and sensitize the tumor to chemical agents 121-123. For instance, Jing Kuang et al. designed an iRGD-modified silica nanoparticles (DOX@MSN-SS-iRGD&1MT) to simultaneously deliver the chemical agent doxorubicin (DOX) and the immune checkpoint inhibitor 1-methyltryptophan (1MT) for anti-glioma treatment (Figure 8) 119. The active targeting iRGD guided the nanoparticles to penetrate across BBB and improved drug accumulation in orthotopic brain tumors. The nanoparticles induced antitumor immune responses and regulated the immunosuppressive microenvironment. The chemo-immunotherapeutic therapy significantly extended the medium survival with a 50% durable cure rate.

**Figure 9:**
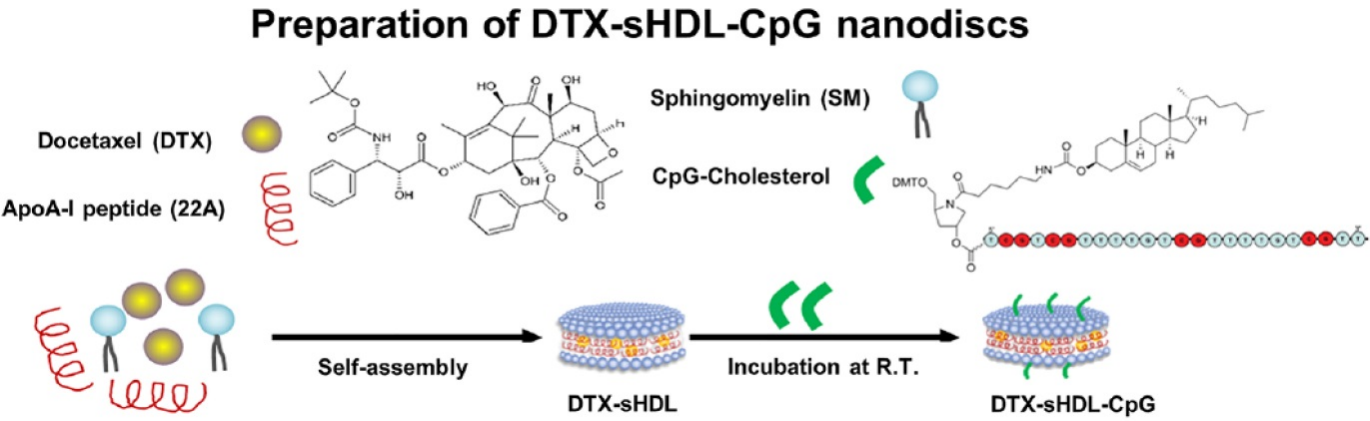
The formulation route of the nanodiscs DTX-sHDL-CpG. Adapted with permission from 125. Copyright 2019 American Chemical Society.

The immunomodulation of the microenvironment of the glioblastoma was another promising approach for tumor regression 124. For example, Padma Kadiyala et al. reported a nanodiscs (DTX-sHDL-CpG) based on high-density lipoprotein (HDL), the synthetic apolipoprotein-I (ApoA-1) 125, which encompassed the anticancer drug docetaxel (DTX) and the immune activator CpG (Figure 9). The nanosystem could elicited antitumor CD8^+^ T cell responses and developed the anti-GBM immunological memory. The DTX-sHDL-CpG showed moderate effect on tumor growth, and the combination of DTX-sHDL-CpG with radiation significantly increased the long-term survival in 80% of GBM-bearing mice.

**Figure 10:**
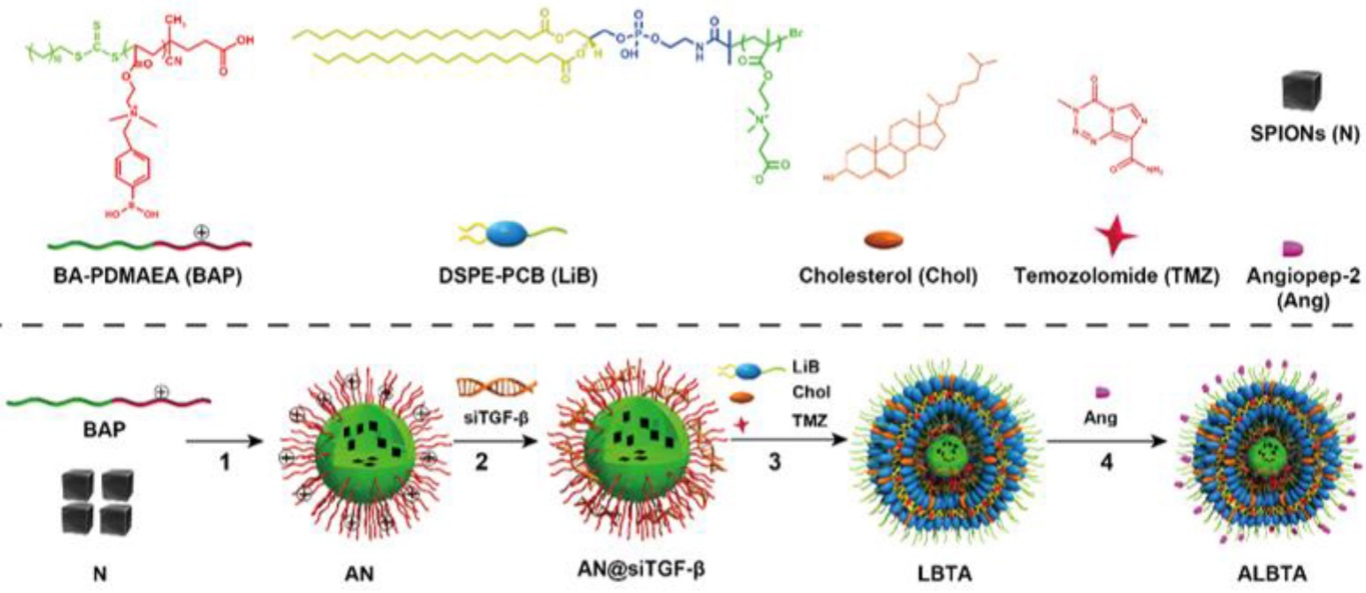
The chemical structural formula of the components and the preparation route of the nanosytem ALBTA. Adapted with permission from 128. Copyright 2018 WILEY‐VCH Verlag GmbH & Co.

Current advances demonstrated that the chemotherapy with temozolomide (TMZ) could induce and aggravate the immunosuppressive tumor microenvironment 126, 127. Regulating the tumor microenvironment by immunotherapy enabled the GBM more sensitive to chemotherapy. Qiao et al. in our team exploited a dual targeting and ROS responsive nanosystem (ALBTA) for immunochemotherapy (Figure 10) 128. SiRNA against tumor growth factor β (siTGF-β) was employed to modify the immune microenvironment of glioblastoma and improve the efficacy of TMZ. To deliver these two drugs in a controlled manner, firstly, ROS-responsive poly[(2-acryloly)ethyl(p-boronic acid benzyl) diethylammonium bromide] (BAP) was chosen to assemble with siTGF-β and triggered to release in cytoplasm of tumor cells. Secondly, zwitterionic lipid distearoyl phosphoethanol-aminepolycarboxybetaine (DSPE-PCB) based envelope (ZLE) was selected to enhance the delivery of TMZ and BAP/siTGF-β (AN@siTGF-β) into cytoplasm. The core-shell structural nanoparticles (ALBTA) significantly improved the immunosuppressive microenvironment *in vivo*, and increased the medium survival time from 19 d (for untreated control group) to 36 d without obvious systemic toxicity in the intracranial glioblastoma mice.

Despite the impressive advances in GBM treatments, the efficacy of immunotherapy still needed further improvements for desirable overall survival. For example, the identification of the specific glioblastoma antigens was required to reinforce the specific immunity responses. Combination of immunotherapy with chemotherapy *via* nanotechnology obviously enhanced the sensitivity of the GBM cells to chemotherapeutic drugs, and regulated the microenvironment of tumors. However, the timing of the immunotherapy for combination 127, 129 and the delivery strategies had critical influences on the therapeutic effects, which needed more efforts to investigate.

### Combined chemotherapy with magnetothermal therapy

Hyperthermia therapy had been developed as a safe and effective complementary therapy for cancer treatments 130. Several magnetic nanomaterials had been used in hyperthermia applications, including iron oxide nanoparticles, cobalt ferrite, iron platinum nanoparticles and so on 131-133. Iron oxide nanoparticles were usually applied as the thermo-agents in cancer treatments and entered into clinical trials 134-136. Since the most thermo-agents were the metal nanomaterials, they were preferable for phagocytose by macrophages rather than the glioblastoma cells. Moreover, the challenges for magnetothermal therapy were the focused heating on the tumor sites with optimum temperature, and not harmed the surrounding normal tissues 137, 138. Hence, nanotechnology was introduced to this therapy for active targeting and inducing more thermo-agents into diseased areas, which promoted the anti-tumor effects 139-141.

**Figure 11:**
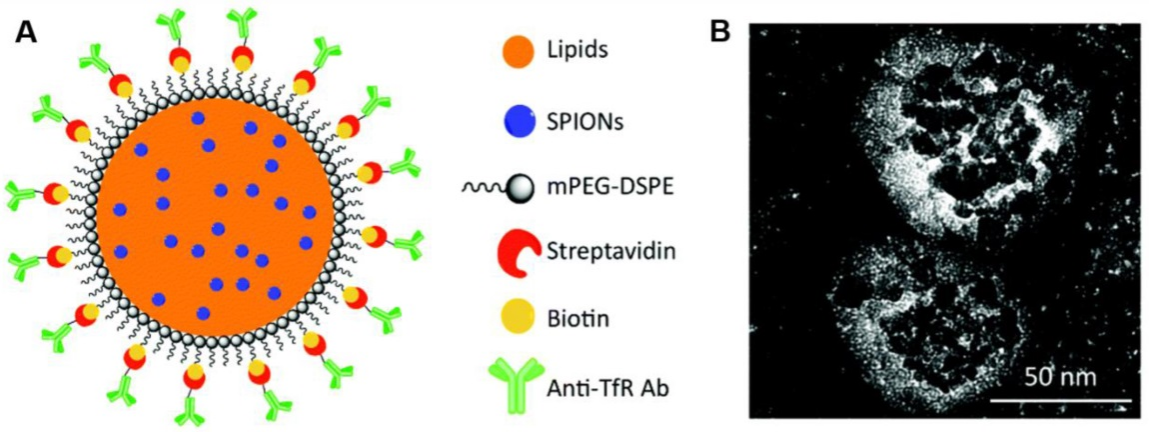
A) The illustration of the lipid magnetic nanovectors (LMNVs) loaded with SPIONs and functionalized with an anti-transferrin receptor antibody (anti-TfR Ab). B) The high magnification transmission electron microscopy (TEM) images of functionalized nanovectors (AbLMNVs). Adopted with permission from 143. Copyright 2019 The Royal Society of Chemistry.

Plenty of evidences showed that the magnetothermal therapy against GBM was more effective by combining with chemotherapy 142. They could enhance the sensitivity of the tumor cells to therapeutic drugs. For example, Gianni Ciofani's group developed a lipid magnetic nanovectors (LMNVs) with 1,2-distearoyl-*sn*-glycero-3-phosphoethanolamine-conjugated poly(ethylene glycol) (DSPE-PEG) (Figure 11) 143, 144. It was functionalized with the antibody against the transferrin receptor (TfR) for dual targeting to endothelial cells of BBB and GBM cells. The superparamagnetic iron oxide nanoparticles (SPIONs) and temozolomide (TMZ) were co-loaded in the lipid nanovectors (LMNVs). The anti-TfR antibodies facilitated targeting to glioblastoma spheroids in multi-cellular *in vitro* models, with 40.5% *vs.* 8.1% for non-target nanovectors. The combined treatment with magnetothermal hyperthermia was able to efficiently disintegrate the GBM spheroids and induce significant tumor cell death. Under magnetic fields, the nanovector induced mild hyperthermia (43°C) and enhanced anticancer effect against U87 MG cells.

**Figure 12:**
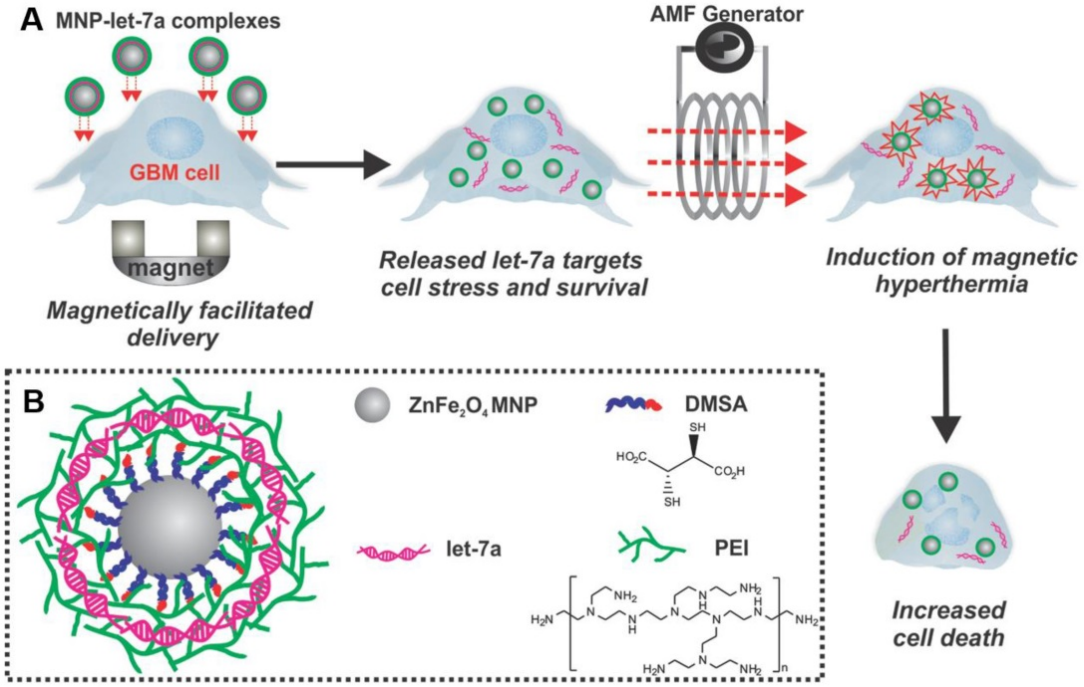
The MNP-PEI/miRNA/PEI complexes enhanced the treatment against brain cancer. A) The MNP complexes were first delivered to GBM cells, which was enhanced by magnetofection. Once inside the cells, let-7a miRNA was released for targeting downstream effectors of HSPs. This sensitized the cancer cells to subsequent magnetic hyperthermia for enhanced apoptosis. B) MNPs were complexed with let-7a miRNA branched PEI *via* a layer-by-layer approach. Adapted with permission from 145. Copyright 2014 WILEY‐VCH Verlag GmbH & Co.

Besides the lipid nanovectors, many synthetic polymeric nanosystems were also exploited as delivery vectors for combined treatments. Yin et al. exploited a highly magnetic zinc-doped iron oxide nanoparticle (ZnFe_2_O_4_) decorated with branched polyethyleneimine (PEI) to deliver lethal-7a miRNA (let-7a) (MNP-PEI/miRNA/PEI complexes), which was a tumor suppressor that inhibited malignant growth by targeting the downstream effectors of heat shock proteins (HSPs) (Figure 12) 145. The nanosystem MNP-PEI/miRNA/PEI exhibited obvious apoptosis of brain cancer cells and led to caspase-3 mediated apoptosis by combining with magnetic hyperthermia.

Wen-Chia Huang et al. reported a tumortropic adipose-derived stem cells (ADSCs) encapsulating the nanotherapeutics (SPNPs) for chemo-thermotherapy 146. The nanotherapeutics were co-assembled with poly(*γ*-glutamic acid-co-distearyl *γ*-glutamate), poly(lactic-co-glycolic acid), the anticancer drug paclitaxel (PTX) and oleic acid-coated superparamagnetic iron oxide NPs. With the guide of ADSCs to tumor sites, hyperthermia was activated by the high frequency magnetic field (HFMF) and triggered drug release in tumor cells. The data *in vivo* demonstrated that the combined therapy extended the lifespan of the brain tumor-bearing mice almost 3 folds, from 11 to 31 days, in comparison with the PBS control group. Whereas, the ADSCs without HFMF activation was only 15 days.

Although the combination of magnetothermal therapy with chemotherapy showed better inhibition against the GBM than single therapy, the combined strategy had not reached the required threshold for efficient clinical applications in GBM treatments. The reasons for this were as follows. Firstly, the magnetothermal efficiency of the magnetic systems was insufficient for clinical applications. The amount and characteristics of magnetic materials and their intratumoral distribution were the key factors for therapeutic effects. Secondly, the timing of the magnetothermal therapy and chemotherapy should greatly influence the overall results of treatments, which needed further investigations. Finally, the design of the delivery systems for the combination was very important, since these would directly affect the anti-tumor effects. Albeit these challenges, such combinations offered multimodal therapeutic strategies in a single treatment. The thermo-agents in the magnetothermal therapy, like the iron oxide nanoparticles, could also exhibit magnetic resonance imaging for tracing the combined nanosystems and monitoring the drug accumulation in *in vivo* distribution.

### Combined phototherapy with immunotherapy

Near-infrared photoimmunotherapy (NIR-PIT) was a recently emerging therapy for cancers 147. It employed a conjugate, which consisted of a near-infrared photosensitizer and a target-specific antibody. They could selectively kill the cancer cells and activate the body's antitumor immune responses. And few of the photo-immunoconjugates displayed toxicity until the activation by the light with specific wavelength. The NIR-PIT was applied for clinical trials against inoperable head and neck cancers by using cetuximab-IR700 (RM1929), and they exhibited promising results 148, 149.

Utilizing the unique character of NIR-PIT, Jing et al. chose the AC133 monoclonal antibody to conjugate with the photosensitizer IR700 (AC133-IR700) for glioblastoma treatments 150. The AC133 could recognize the CD133 on human cancer stem cells, and enhance the active targeting to orthotopic AC133^+^ brain tumor. The treated group extended the median survival time to 33.5 days in comparison with non-irradiated control group (with 15 days of survival time). The epidermal growth factor receptor (EGFR) was the general genetic mutation in GBM. Hence, an EGFR-specific antibody (Z_EGFR-03115_) was conjugated to the photosensitizer IR700DX for glioblastoma treatments 151. The tumor distribution of the Z_EGFR-03115_-IR700DX was 6-fold higher than that of the control group Z_Taq_-IR700DX. And they exhibited a significant inhibition of tumor growth in U87-MGvIII bearing mice over a period of 18 days.

**Figure 13:**
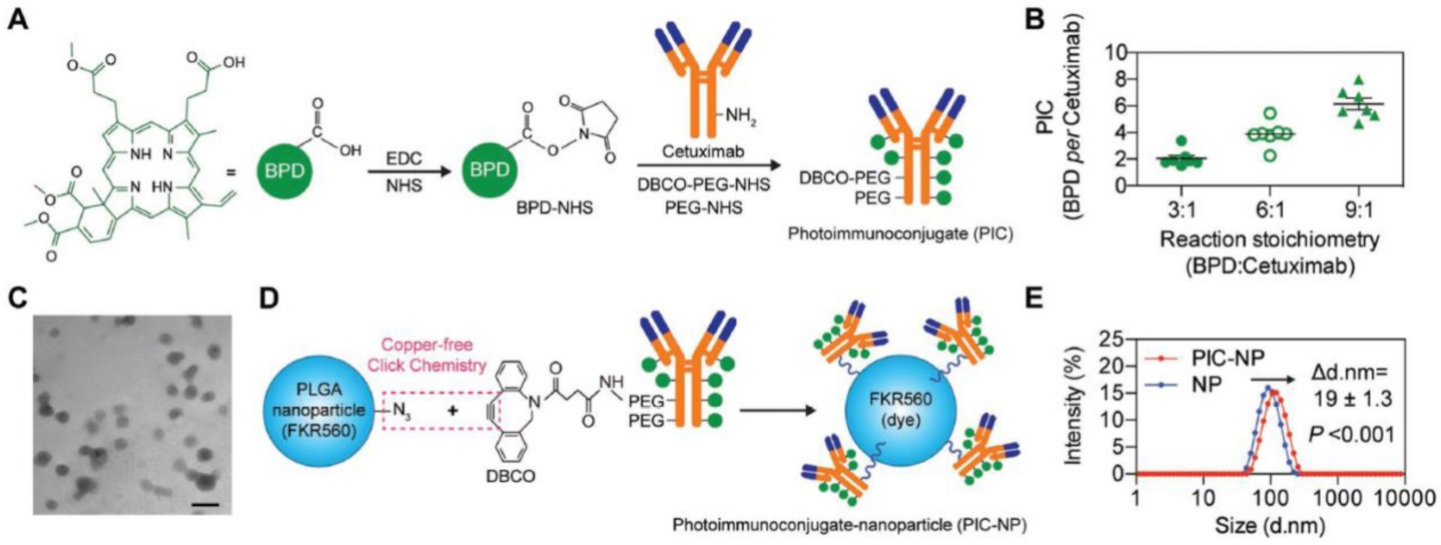
The synthesis route of photo-immunoconjugate nanoparticles (PIC-NPs). A) The illustration of PIC synthesis. Benzoporphyrin derivative (BPD) photosensitizers were conjugated to PEGylated cetuximab *via* carbodiimide crosslinker chemistry. B) The varied stoichiometry of BPD reacted with cetuximab. C) Transmission electron microscopy (TEM) image of poly(ethylene glycol)-poly(lactic-co-glycolic acid) (PEG-PLGA) polymeric nanoparticles prepared *via* nanoprecipitation method. Scale bar 100 nm. D) Schematic depiction of PIC-NP synthesis *via* copper-free click chemistry. Azide-containing FKR560 dye-loaded PLGA nanoparticles were reacted with the dibenzocyclooctyne (DBCO)-containing PICs to form PIC-NPs. E) Covalent conjugation of PICs onto 80 nm PEG-PLGA NPs to form monodispersed PIC-NPs around 100 nm in diameter. Adapted with permission from 152. Copyright 2018 WILEY‐VCH Verlag GmbH & Co.

However, due to the limited ratio of the photosensitizer-to-antibody, the amount of photo-immunoconjugates delivered to tumor cells was not sufficient. Hence, Huang et al. adopted poly(lactic-co-glycolic acid) (PLGA) nanoparticles to overcome the obstacles 152. The photo-immunoconjugates benzoporphyrin derivative (BPD)-cetuximab was bound to PLGA nanoparticles by click coupling (Figure 13). The photo-immunoconjugate nanoparticle (PIC-NP) could significantly enhance the photosensitizers to cancer cells, and increase light-activated cytotoxicity in EGFR-overexpressing U87 cells. The nanotechnology improved the overall treatment outcomes comparing with the photo-immunoconjugates.

Compared to classic photodynamic therapy, the photoimmunotherapy utilized the specific antibodies that could facilitate targeting the tumor cells without damaging the normal cells. It also enhanced photosensitizer delivery into tumors and increased the light-activated cytotoxicity. Despite these promising advances, the limited ratio of photosensitizer-to-antibody restricted the amount of photosensitizers delivered by photo-immunoconjugates. Hence, the nanodelivery systems provided the larger loading capacity and increased accumulation in the tumors. They efficiently maximized the photo-toxicity to the targeted tissues. However, the photoimmunotherapy for glioblastoma was still in its infancy. The immune activation was not studied well for these anti-glioma treatments.

### Combined gene therapy with immunotherapy

Gene therapy had emerged as a novel treatment for various human diseases including cancers. It could specifically regulate the oncogenes in the anti-tumor treatments 153, 154. The adenovirus-mediated gene therapy with sitimagene ceradenovec and ganciclovir after resection increased survival time of patients with newly diagnosed glioblastoma multiforme 155. Hence, the gene therapy was usually proposed as a useful adjuvant for the current glioblastoma treatments 156. The combination of gene therapy with immunotherapy should increase the whole outcomes of glioblastoma treatments 157, 158. For example, Maria-Carmela Speranza et al. utilized the nonreplicating adenovirus containing the HSV TK gene (AdV-tk) to potentiate the anti-PD-1 efficacy in syngeneic glioblastoma mouse models. The AdV-tk could upregulate the IFN signaling and increase the PD-L1 levels. And cytotoxic CD8^+^ T cells were induced to accumulate in tumors. The combination treatment significantly increased the percentage of long-term survival (LTS≥100 days) animals from 30-50% (single agents) to 88% 159.

**Figure 14:**
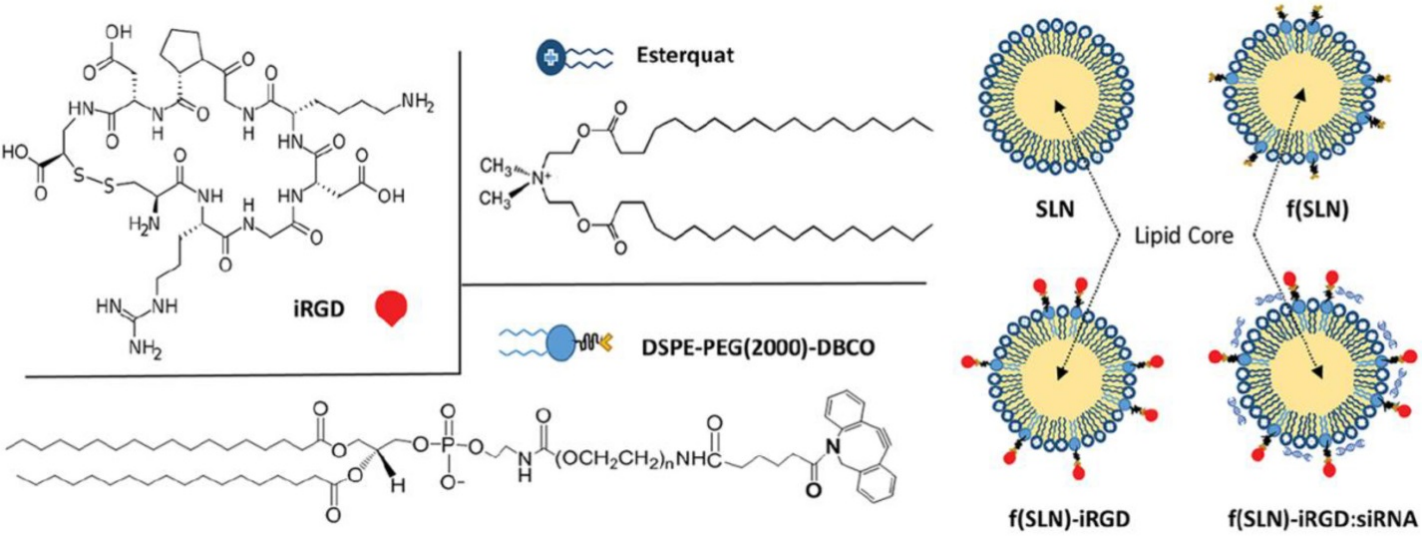
The chemical structures of the DSPE-PEG(2000)-DBCO, Esterquat and iRGD peptide, and the construction of the nanoparticles SLN, f(SLN), f(SLN)-iRGD and f(SLN)-iRGD:siRNA. Adapted with permission from 163. Copyright 2019 American Chemical Society.

Despite the potentials of gene therapy, the intrinsic characteristics of the gene limited the efficient delivery to tumor sites and hindered their progress to the clinical applications 26, 160, 161. Especially the crossing through the BBB exhibited an extra challenge for anti-glioma treatments. Nanoparticles could compensate the shortages of the gene and safely deliver gene and immunotherapeutic agents to brain tumors 162. For instance, Gulsah Erel-Akbaba et al. developed a cyclic peptide iRGD (CCRGDKGPDC)-decorated solid lipid nanoparticle (SLN) to deliver siRNA against both the epidermal growth factor receptor (EGFR) and programmed cell death ligand-1 (PD-L1) for combined therapy (Figure 14) 163. The targeting nanoparticles f(SLN)-iRGD:siRNA led to 54.7% and 58.6% decrease for EGFR and PD-L1, respectively. Moreover, the median survival of the mice treated with f(SLN)-iRGD:siRNA with radiation increased to 38 days. These three combinations largely improved the survival of the GL261-bearing mice.

Evidences exhibited that the gene therapy was usually applied to regulate the immunosuppressive signals and enhance the systemic therapy in glioma treatments 164. These could synergistically augment the immune responses with immunotherapy. But the reported studies on this strategy were less. The gene therapy and immunotherapy usually served as adjuvant treatments in existing anti-glioma therapies. Moreover, the lack of specific genes and antigens was still the critical barriers for satisfied treatments.

## Conclusion and Perspective

Glioblastoma is a complex and deadly disease in central nervous system. The high infiltration, heterogeneity, rapid growth rate and regeneration ability of the GBM stem cells result in poor prognosis and high recurrence. Furthermore, the existence of the blood-brain barrier (BBB) greatly hinders adequate accumulation of the therapeutic drugs in tumor sites, which also induces drug-resistance to therapy. With the deep understanding of glioblastoma, improved therapeutic strategies are exploited to enhance the efficacy against glioblastoma. They mainly include chemotherapy, gene therapy, radiotherapy, immunotherapy, phototherapy and magnetothermal therapy. Considering the intricate characteristics of GBM, combined-therapeutic strategies are developed for synergistic anti-glioma effects and reduced drug-resistance 165. And the pros and cons of the combined therapies are summarized and illustrated in Table 1.

In review of the intensive studies for GBM, some problems should be settled for optimizing GBM treatment. Firstly, the choices of the therapeutic agents in combined strategies are very important for overall treatment outcomes. The therapeutic mechanisms of the agents should not counteract the pharmaceutical effects for each other. Some of the combinations in clinical trials do not obtain the satisfied results compared with monotherapies 40, 166. It has been reported that the combination of erlotinib and sorafenib in phase II study exhibits significant pharmacokinetic interactions, which may display negative impacts on the therapeutic efficacy 167. Moreover, the recent therapeutic agents selected for therapy can not cover the critical points in the progression of GBM. Therefore, multi-modal combined strategies should be tesed in anti-glioma treatments. Secondly, the timing of the therapeutic strategies in combination needs more investigations. For instance, the administration timing of chemotherapy in combinations obviously affects the efficacy of immunotherapy 127. Hence, alternative therapeutic strategies are required to attempt for maximizing the therapeutic effects. Finally, the suitable delivery systems are demanded for effective drug delivery. Increasing studies demonstrate that nanosystems perform preferable therapeutic results in comparison with free therapeutic agents. The delivery platforms possess active brain targeting and diverse stimuli-responses 168, 169. Therefore, these strategies significantly promote the loading agents to cross BBB and accumulate in GBM cells. And they extend the overall survival time of GBM in animal experimental studies. However, glioblastoma remains a complicated cancer. The current reported targets are not specific for the endothelial cells of BBB and tumor cells. The further and better understanding of the intricate tumor microenvironment is still required. Hence, the specific targets and the suitable stimuli-responses of the GBM are required to exploit, which will facilitate the diverse agents to release in the right places and ensure sufficient therapeutic agents in GBM cells. Moreover, most of the biomaterials in studies have not been approved by FDA, which aggravate the difficulties for clinical translation.

Although the current combined-therapeutic strategies have enhanced the efficacy of GBM treatments, great efforts are still required on the understanding the complicated mechanisms of the growth, progression and invasion process of GBM. Multidisciplinary knowledge of GBM will guide the development of optimal therapeutic strategies and rational nanosystems for satisfied outcomes, and direct the investigation for other malignant diseases in central nerves system.

## Figures and Tables

**Table 1:** The pros and cons of the combined treatments against glioblastoma.

Combined treatments	Advantages	Disadvantages
Chemotherapies	Reduced drug resistance; Multiple mechanisms	Non-specificity to tumor cells
Chemotherapy and radiotherapy	Increased tumor sensitivity to chemotherapeutic drugs	Hard to control radiation margin; Timing of radiotherapy
Chemotherapy and phototherapy	Multiple mechanisms	Limited tissue penetration of light; Drug to light interval
Chemotherapy and immunotherapy	Multiple mechanisms; Reduced metastasis and recurrence	Lack of specific antigens; Timing of immunotherapy
Chemotherapy and magnetothermal therapy	Multimodality; Theranostics	Thermal efficiency; Hard to focus heating on tumor sites
Phototherapy and immunotherapy	Enhanced targeting; Reduced metastasis and recurrence	Restricted ratio of photosensitizer to antibody;
Gene therapy and immunotherapy	Indirect anti-tumor effect; Reduced drug resistance	Lack of specific genes and antigens
